# Introduction to the Special Issue: Assessing the Environmental Adaptation of Wildlife and Production Animals: Applications of Physiological Indices and Welfare Assessment Tools

**DOI:** 10.3390/ani10122280

**Published:** 2020-12-03

**Authors:** Edward Narayan

**Affiliations:** 1 School of Agriculture and Food Sciences, Faculty of Science, The University of Queensland, St Lucia, QLD 4072, Australia; e.narayan@uq.edu.au; Tel.:+61-7-5460-1693; 2 Queensland Alliance for Agriculture and Food Innovation, The University of Queensland, St Lucia, QLD 4072, Australia

Wild animals under human care as well as domesticated farm production animals are often exposed to environmental changes (e.g., capture and transportation). Short-term or acute changes in physiological indices (e.g., heart rate, respiration, body temperatures, immune cells and stress hormonal biomarkers) provide crucial information regarding the responses of animals to novel environments, and they could provide crucial determining factors for evaluating the long-term health and welfare of animals. The goal of this special issue is to provide examples of new research and techniques that can be used to monitor short- and long-term environmental adaptation of animals under human care. Examples of research include applications of physiological indices and welfare assessment methods (e.g., morphological and morphometric data, behavioural assessments, thermal profiles and physiological markers) in any wildlife or production animal (e.g., rescued and rehabilitating animals, pets, competition animals, farm animals and zoo animals), in response to environmental and management-related factors.

This book is a reprint of the papers published in the Special Issue: “Assessing the Environmental Adaptation of Wildlife and Production Animals: Applications of Physiological Indices and Welfare Assessment Tools”.

Chapter 1—This research was based on a retrospective analysis of clinical data and characterises this based on categories of stress experienced by avian wildlife patients. It demonstrated the factors associated with urbanization, which exposes avian wildlife to an array of environmental stressors that result in clinical admission and hospitalisation. It showed that the most common outcome of avian patients that suffered from vehicle-related injuries or other impact injuries was euthanasia. Immobility and abnormal behaviour were the most commonly occurring primary stressors of avian patients. Finally, trauma and fractures were the most common occurring secondary stressors in avian patients. The most common outcome of all these stressors was euthanasia. This study also provided a categorisation system for the stressors (preliminary, primary and secondary) that may be used to monitor the stress categories of wildlife patients and gain a deeper understanding of the complex notion of stress.

Chapter 2—Artificial insemination programs are used to improve reproductive output in livestock animals. This study showed the possible influence of ejaculation collection in breeding boars on their oxytocin profiles. Using saliva collection, the research measured total (protein bound) and free oxytocin in pigs. Research showed that ejaculation influences the salivary oxytocin concentrations in breeding boars, although this influence varies according to age, libido and breed.

Chapter 3—Pharmacological and biological validation are important for establishing minimally invasive stress hormone evaluation in animals. Here, researchers were able to validate two faecal glucocorticoid enzyme immunoassays (faecal corticosterone metabolites-FCMs, EIAs; a corticosterone EIA, and a group-specific 5α-pregnane-3β,11β,21-triol-20-one EIA) in deermice (*Peromyscus maniculatus*) by challenging individuals with dexamethasone and adrenocorticotropic hormone (ACTH). Researchers discuss the need for careful physiological/biological validation of assays prior to applications in animal studies.

Chapter 4—Veterinary intervention is an important aspect of wildlife rescue and rehabilitation programs. In this research case study on the roe deer (*Capreolus capreolus*) from Italy, researchers identified a potential biomarker (whole blood lactate concentrations) as an early indicator of lactataemia status to predict the outcome of clinical intervention. Biomarkers such as these should be used in conjunction with clinical veterinary intervention to provide appropriate care and outcome decisions for rescued wildlife.

Chapter 5—Quantification of acute stress is an important component of wildlife management and care in zoos. In this research, the investigators compared the glucocorticoid concentrations in response to various types of potential stressors present during the standard operation of a temporary housing facility between three species, namely, ring-tailed lemurs, collared brown lemurs and white-headed lemurs. Researchers employed polyclonal antibodies directed against the metabolite 11-oxo-etiocholanolone I. Researchers found some species-related differences in the physiological stress responses of the lemurs, however, the general patterns across treatments were similar, but individual reproductive status may also influence the stress responses in grouped housing situation.

Chapter 6—Wildlife hunting is an example of intensive human–animal interaction which can generate physiological stress in animals. Red deer (*Cervus elaphus*) is the target of intensive seasonal hunting in the Lousã Mountain region, Portugal. Using a combination of sample types (blood, faeces and hair), researchers in this study quantified glucocorticoid levels in red deer across hunting seasons. The researchers discuss the applications of specific sample types for evaluating acute and chronic stress responses of red deer in the hunting season.

Chapter 7—Tigers (*Panthera tigris*) are an endangered species and it is crucial to obtain vital health indices of tigers for rescue, rehabilitation and captive management programs. In this pilot study, the researchers demonstrated the application of serum protein electrophoresis as a useful tool in monitoring the health of tigers.

Chapter 8—Stress endocrine response can influence immune response in animals. Similarily, immune response biomarkers can be used to index stress responses in animals. For example, salivary immunoglobulin A (sIgA) has been proposed as a potential indicator of welfare for various species, including Asian elephants, and may be related to adrenal cortisol responses. Here, researchers distinguished circadian rhythm effects on sIgA in male and female Asian elephants and compared patterns to those of salivary cortisol, information that could potentially have welfare implications. Researchers discovered a daily quartic pattern of SIgA in elephants, which can be important information for designing future experiments to standardize field data collection.

Chapter 9—Wild mammals can be highly cryptic and there remains substantial knowledge gaps regarding the physiological control of reproduction in species, such as red deer (*Cervus elaphus* L., 1758). Researchers evaluated the concentration of cortisol and progesterone, extracted from blood and hair, in 10 wild and pregnant red deer females. Researchers successfully quantified cortisol and progesterone in hair samples obtained from the hinds of deer sampled during a regional selective hunting plan. It may be possible the deer were actively breeding during the sampled period and the methods could be used to evaluate the reproductive ecology and welfare of deers used for human hunting purposes.

Chapter 10—The current study developed a baseline welfare assessment protocol for captive Punjab urial adapted from the welfare protocol for domestic sheep from the Welfare Quality^®^ project. It was able to apply the protocol for Punjab urial across two facilities and provided recommendations for areas of improvement for captive management and breeding.

Chapter 11—Koalas (*Phascolarctos cinereus*) are Australia’s iconic marsupial species and they face heightened threats from anthropogenic induced environmental change. In this study, the researchers validated the application of a thermal imaging technology (FLIR530TM IR thermal imaging camera) to evaluate heat signatures in koalas in a captive zoo housing facility. The study discussed the technical limitations and applications of this method for tracking heat stress in koalas in zoos.

Chapter 12—Wildlife are commonly impacted by parasites from their surroundings and they can also serve as hosts for zoonotic pathogens. Parasites activate immune responses of wildlife which can be quantified using morphological, blood and tissue analysis. Here, the researchers evaluated the relative impact of parasite pressure vs. parasite load on different host species, using bank voles (*Myodes glareolus*) and wood mice (*Apodemus sylvaticus*) as study species. The researchers sampled sub-adult males to quantify their immune function, infestation load for ectoparasites and gastrointestinal parasites, and infection status for vector-borne microparasites. They used regression trees to find out whether variation in immune indices could be explained by among-site differences (parasite pressure), among-individual differences in infestation intensity and infection status (parasite load) or other intrinsic factors. The research outcome showed that both parasite pressure and parasite load influence the immune system of wild rodents.

Chapter 13—It is important to validate minimally invasive stress hormone assays for each species due to potential species-specific differences in metabolism and excretion of steroids. In this study, the researchers physiologically validated a faecal cortisol metabolite (FCM) enzyme-immunoassay for male reindeer. Researchers conducted a physiological validation of an 11-oxoaetiocholanolone enzyme immunoassay (EIA) for measuring faecal cortisol metabolites (FCMs) in male reindeer by the administration of an adrenocorticotrophic hormone. Researchers also identified the faecal samples belonging to individual animals using DNA analysis across time. This study reports a successful validation of a non-invasive technique for measuring stress in reindeer, which can be applied in future studies in the fields of biology, ethology, ecology, animal conservation and welfare.

Chapter 14—Minimally invasive hormone monitoring methods can be used to evaluate the physiological responses of aquatic fish species to habitat quality. Here, the researchers validated mucous cortisol assays in wild freshwater fish (Catalan chub, *Squalius laietanus*) living across a pollution gradient. They compared the mucous cortisol levels with cortisol levels in blood and haematological parameters. The results showed that the variation in cortisol in skin mucus followed a similar pattern of response to that detected by the quantification of cortisol levels in blood and the hematological parameters, such as erythrocytic alterations and neutrophil to lymphocyte ratios. Skin mucus could be potentially used as a biomarker in fish welfare evaluation.

